# Supplementary material for: Evolution of protease activation and specificity via alpha-2-macroglobulin-mediated covalent capture
Source: Nat Commun. 2023 Feb 11;14:768. doi: 10.1038/s41467-023-36099-7 (PMC9918453; doi:10.1038/s41467-023-36099-7)
Supplement: Supplementary file 1 — Supplementary Information [file 41467_2023_36099_MOESM1_ESM.pdf]

Supplementary Information

Evolution of protease activation and specificity via alpha-2-macroglobulin-mediated covalent capture

Author list:

Philipp Knyphausen[1,3], Mariana Rangel-Pereira[1], Paul Brear[1], Marko Hyvönen[1], Lutz Jermutus[2] and Florian Hollfelder*[1]

Affiliation:

[1]Department of Biochemistry, University of Cambridge, 80 Tennis Court Road, CB2 1GA, Cambridge, UK.

[2] Research & Early Development, Cardiovascular, Renal & Metabolism, BioPharmaceuticals R&D, AstraZeneca, 1 Frances Crick Avenue, CB2 1GA, Cambridge, United Kingdom.

Current address:

[3]Gene Editing Discovery and Engineering, Genomic Medicine, Bayer AG, Nattermannallee 1, 50829 Cologne, Germany.

*Correspondence: fh111@cam.ac.uk (F. Hollfelder).

@Twitter: @hollfelderlab

Lab website: https://www.bioc.cam.ac.uk/hollfelder

Orcids:

PK: 0000-0002-2544-7920
LJ: 0000-0001-7660-8467
MH: 0000-0001-8683-4070
FH: 0000-0002-1367-6312

Table of contents

	Page
**(A) Supplementary Figures**	S3
Supplementary Figure 1:<br>Developing A2M^{cap} for the detection of SplB activity	S3
Supplementary Figure 2:<br>Characterisation of clones from selection of pre-SplB error-prone PCR library	S4
Supplementary Figure 3:<br>Comparison of B-factors of Spl structures	S6
Supplementary Figure 4:<br>A2M^{cap}-based Spl substrate profiling	S7
Supplementary Figure 5:<br>Details of SCHEMA shuffling library generation	S8
Supplementary Figure 6:<br>Shuffling library selection	S9
**(B) Supplementary Tables**	S10
Supplementary Table 1:<br>Theoretical molecular weights of SplB WT and mutants	S10
Supplementary Table 2:<br>Crystallographic data summary for 6YV5 and 6YV6	S11
Supplementary Table 3:<br>DNA constructs used in this study	S12
Supplementary Table 4:<br>DNA oligonucleotides used in this study	S13
Uncropped scans of all and gels	S14

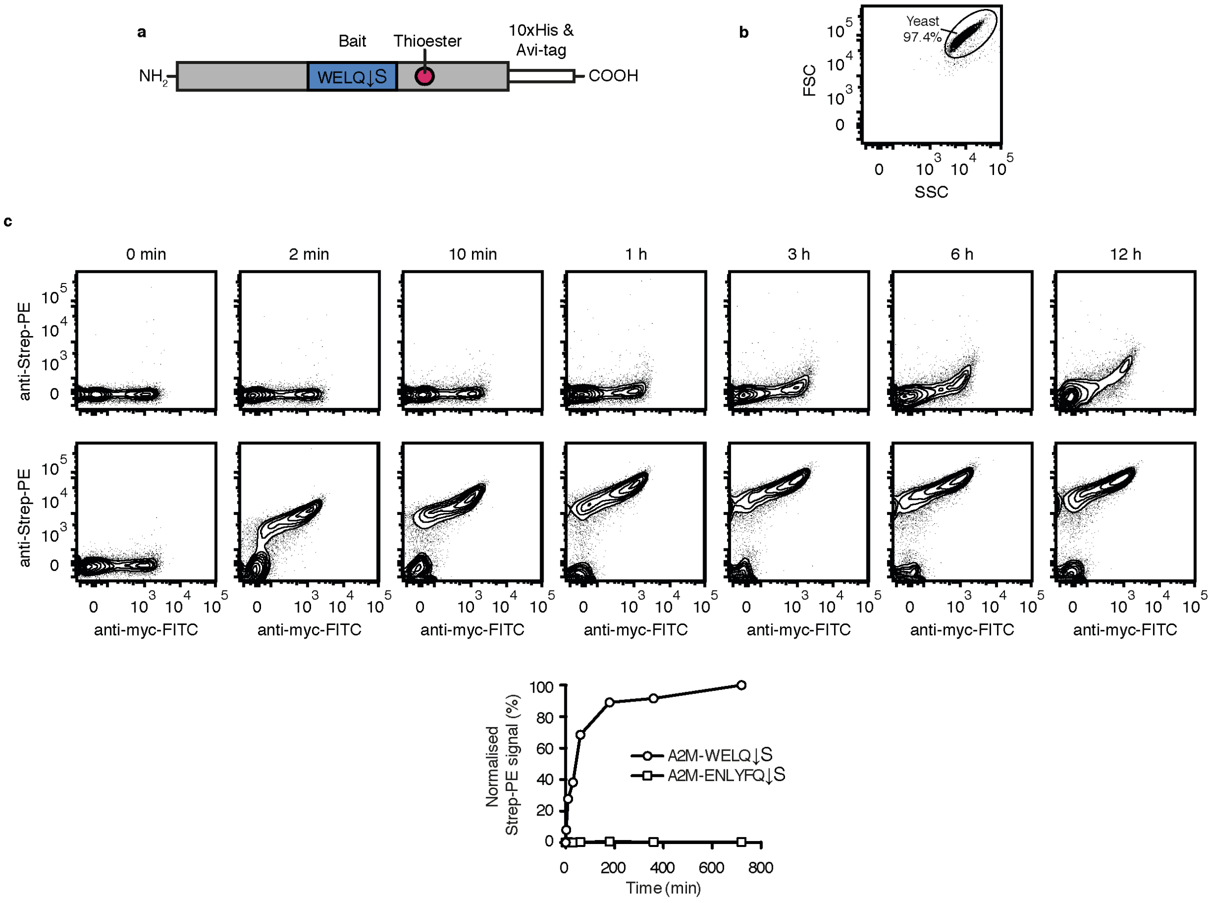

Supplementary Fig. 1: Developing A2M^{cap} for the detection of SplB activity.
(a) Overview of the A2M capture substrate expressed in *E.coli,* which is then used for protease activity detection. A C-terminal Avi-tag and 10x Histidine (His)-tag enable *in vivo* biotinylation and Ni-NTA affinity chromatography-based purification, respectively. The WELQ↓S sequence is the SplB recognition sequence and replaces the native A2M bait sequence. **(b)** Exemplary gating strategy for the flow cytometry of yeast cells. **(c)** Time course flow cytometry measurement of *S. cerevisiae* EBY100 cells displaying SplB, probed with the indicated A2M substrate and subsequently fluorescently labelled with a Streptavidin-PE (for biotinylated A2M) and anti-myc-FITC (for myc-tagged SplB). *n*=1 flow cytometry measurement, source data is provided as a Source Data file for panel c. FITC = Fluorescein isothiocyanate, Strep-PE = Streptavidin-Phycoerythrin

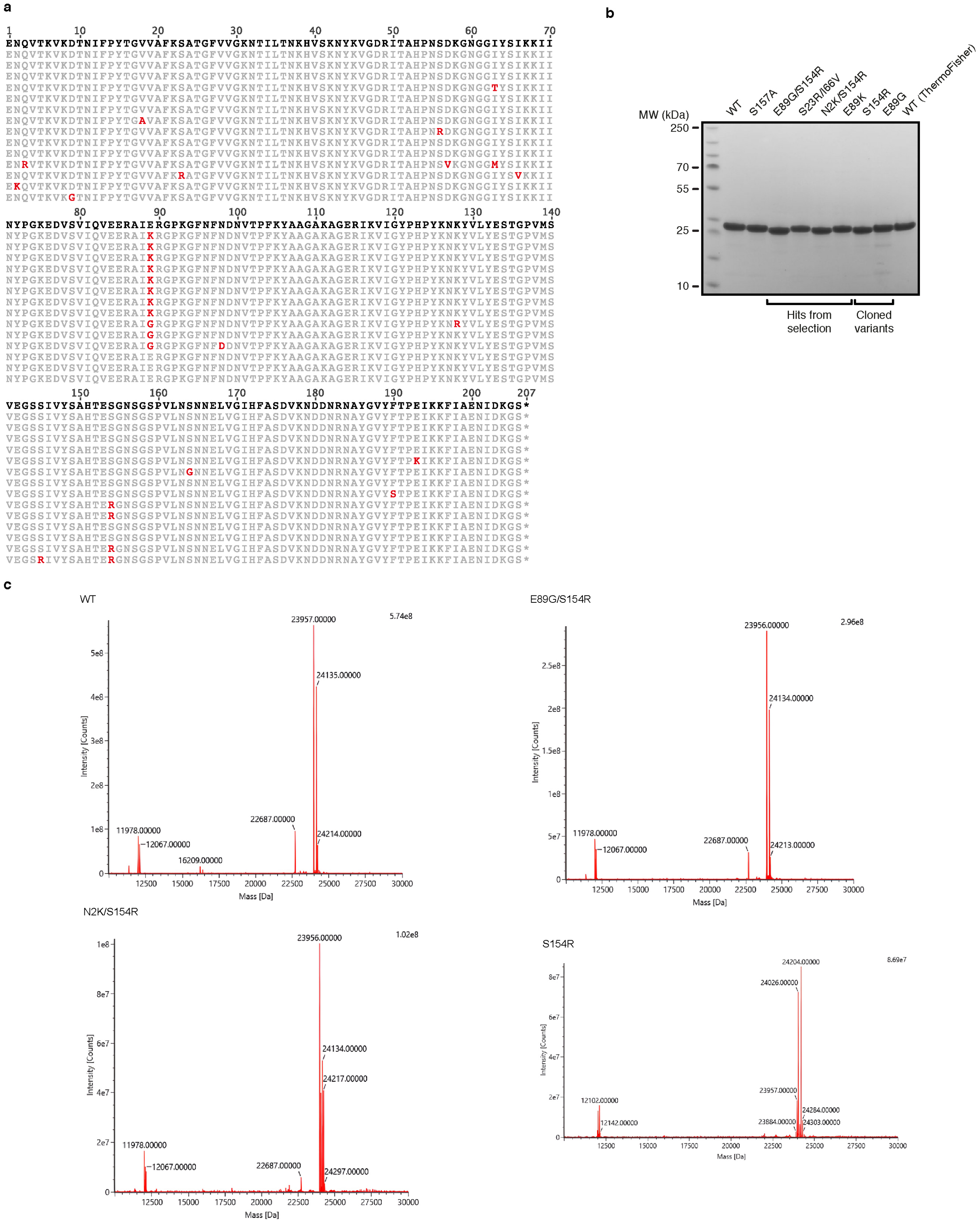

Supplementary Figure 2: Characterisation of clones from selection of pre-SplB error-prone PCR library.

(a) Amino acid sequences of 14 clones picked after two rounds of selection and regrowth (obtained via Sanger sequencing). **(b)** SDS-PAGE of *E. coli*-purified SplB variants compared with commercially supplied wildtype SplB. ‘Hits from selection’ are mutants directly obtained from the selection of the error-prone library. ‘Cloned variants’ are two single mutations generated for individual testing (result of *n*=1 protein purification). **(c)** LCMS (ESI-TOF) mass spectrometry of purified SplB WT and mutants. The observed base peaks correspond to pre-SplB without the first methionine (that is presumably lost in the gas phase) rather than processed SplB. The analysis was carried out on the mass spectrometer Vion IMS-QTOF coupled with a UPLC system (column: Acquity UPLC Protein BEHC4 with dimensions 2.1 mm x 50 mm). At a flow rate of 0.2 mL/min mixtures of two buffers, A and B, were used to elute the

sample (A: water with 0.1% formic acid; B: acetonitrile): 0-1 min: in a A:B ratio of 95:5; 1-5 min: gradient between 0:100, 5-6 min: 0:100; 7 min-end: 95:5. The data were processed by UNIFI software that controls LCMS analysis and runs the Vion spectrometer.

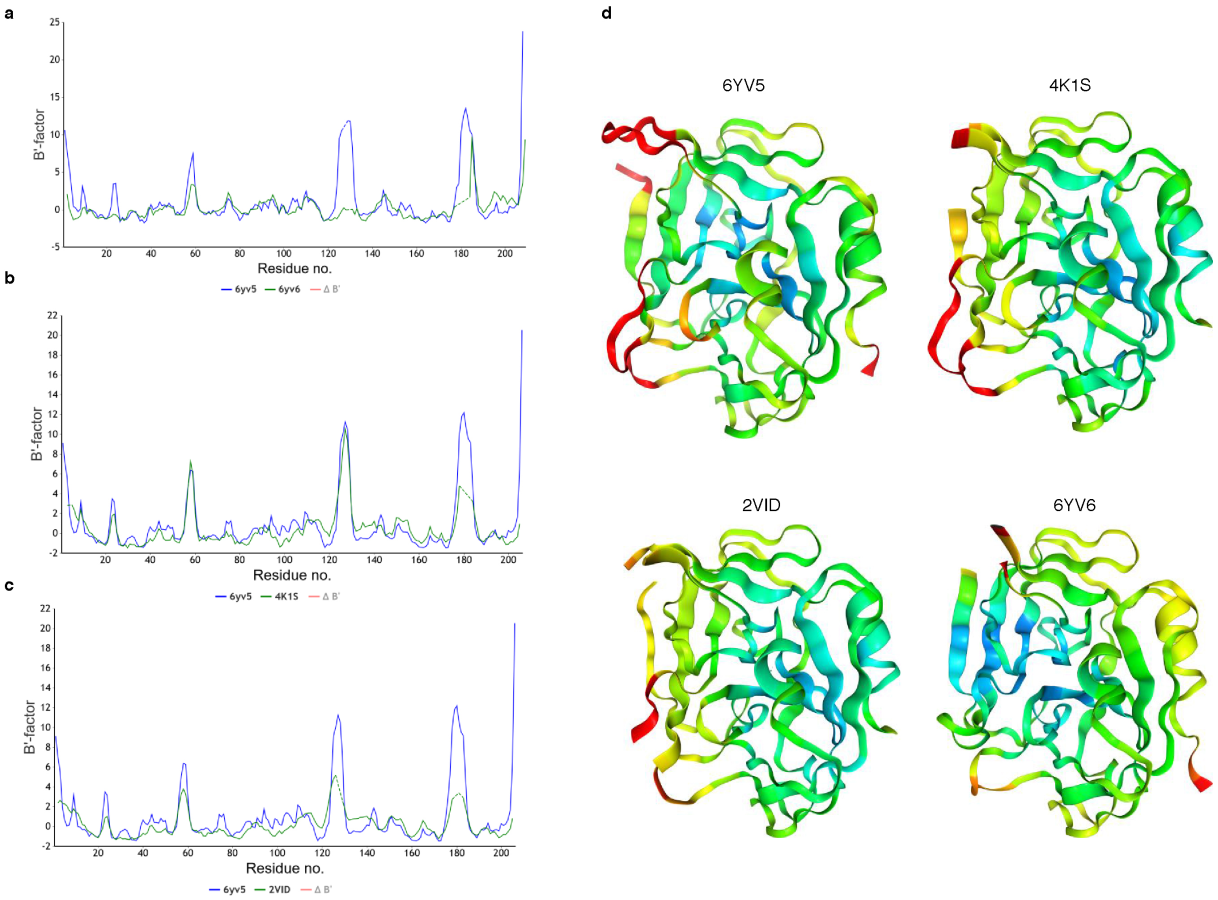

Supplementary Figure 3: **Comparison of B-factors of SpI structures.**

(a-c) B-factors of the structures described here (PDB:6YV5 and 6YV6) and previously determined structures of activated SplB (PDB:2VID) and pre-SplB (PDB:4K1S) were normalised using the BANΔIT server (https://bandit.uni-mainz.de/to) to take into account different overall B-factors in the structures. The resulting B'-factors are plotted in each graph for 6YV5 and each other structure, showing a similar overall pattern of variation, with the most significant differences readily explained by differential crystal contacts. **(d)** The structures of 6YV5, 4K1S, 6YV6 and 2VID are shown as ribbon diagrams coloured according to their B'-factors.

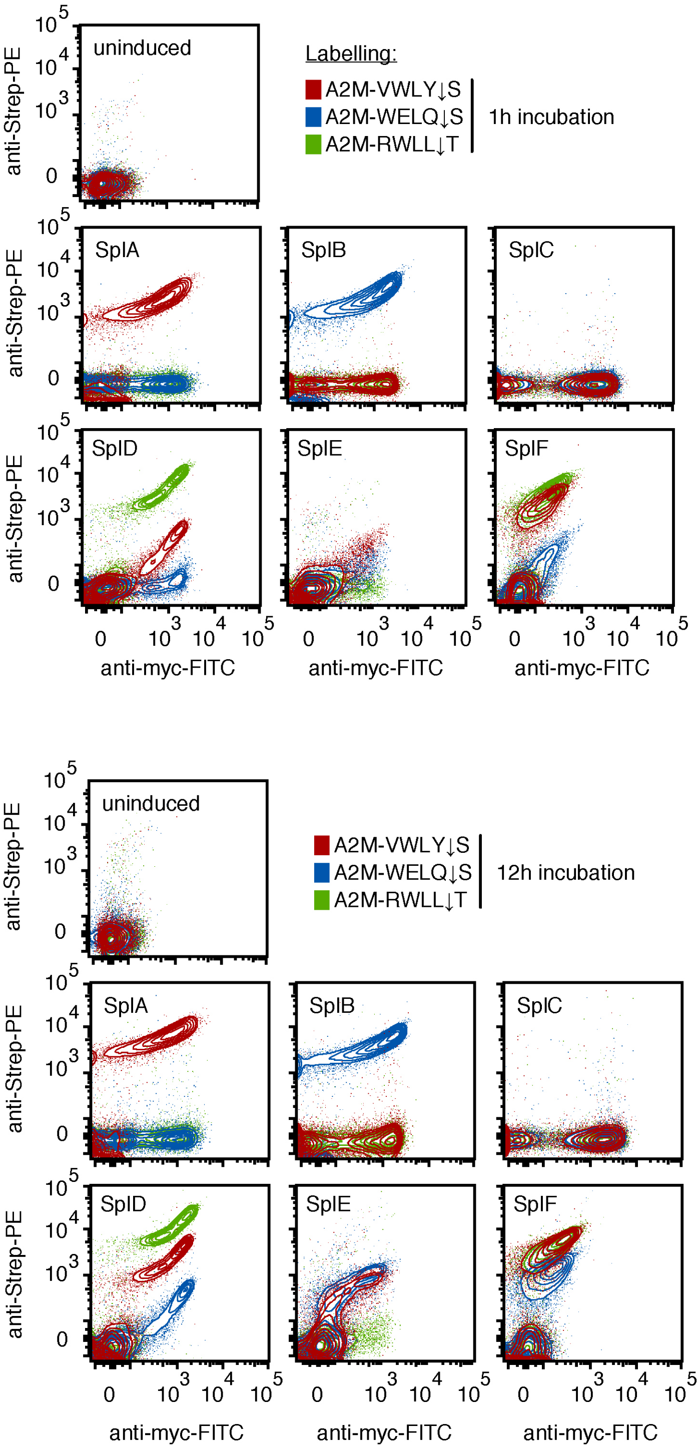

Supplementary Figure 4: A2M^{cap}-based Spl substrate profiling.
Flow cytometry of Spl protease-displaying *S. cerevisiae* cells probed with substrates A2M-VWLY↓S, -WELQ↓S and -RWLL↓T. *Top panel*: 1h incubation time; *bottom panel*: 12h incubation time (Compare with Fig. 4a, representative data of *n*=2 independent inductions and flow cytometry measurements). FITC = Fluorescein isothiocyanate, Strep-PE = Streptavidin-Phycoerythrin. Source data are provided as a Source Data file.

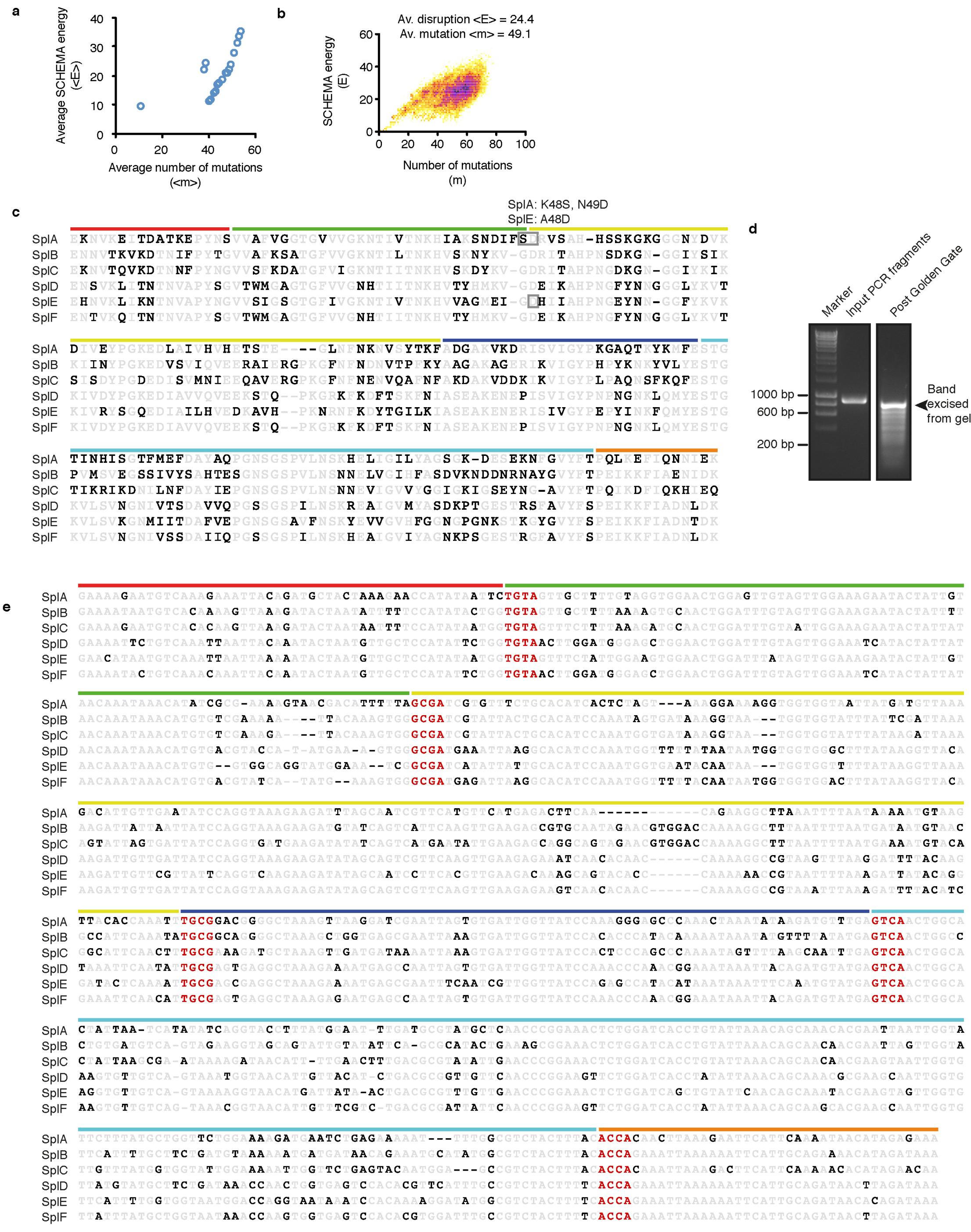

Supplementary Figure 5: Details of SCHEMA shuffling library generation.
(a) SCHEMA algorithm output for a 6 fragment library (with 5 crossovers) of SplA-F. Each dot represents the average E and m for a set of cross-over points. **(b)** SCHEMA Energy and mutation profile of the selected cross-over points with the average value for each stated above the plot. **(c)** Amino acid alignment of SplA-F with SCHEMA blocks highlighted by color. Mutations that were introduced into SplA and SplE to enable common Golden Gate overhangs are indicated. **(d)** Agarose gel of the pool of Spl PCR fragments before and after Golden Gate-based shuffling (*n*=1 cloning attempt for the shuffling library). **(e)** Nucleotide alignment of SplA-F with SCHEMA blocks highlighted by color. Golden Gate overhangs are indicated in red.

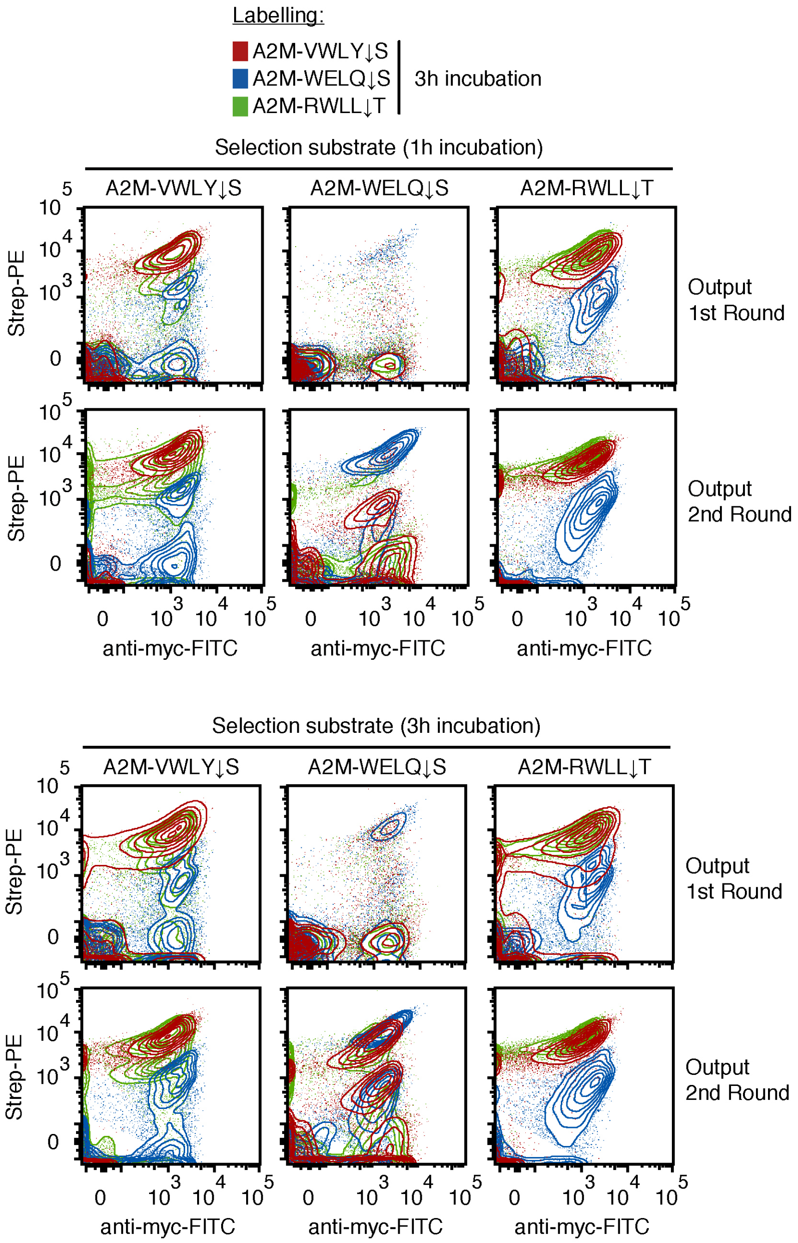

Supplementary Figure 6: Shuffling library selection.
Flow cytometry of input and output populations probed with the three different A2M substrates used during selection. Top panel: Selection over two rounds using 1h incubation for the each indicated substrate. Bottom panel: Selection over two rounds using 3h incubation for the each indicated substrate. The labelling was done separately with each substrate with incubation time of 3h. (Compare with Fig. 5c, representative data of *n*=2 independent inductions and flow cytometry measurements for 1st round and input library, n=1 for 2nd round). FITC = Fluorescein isothiocyanate, Strep-PE = Streptavidin-Phycoerythrin. Source data are provided as a Source Data file.

(B) Supplementary Tables

Supplementary Table 1: Theoretical and observed molecular weights of SplB WT and mutants

**SplB variant***	**pre-SplB[a] (Da)**	**pre-SplB w/o first methionine (Da)**	**processed SplB[b] (Da)**	**Observed peaks[c] (Da)**	**Abs. difference observed vs. calculated molecular weight[d] (Da)**	
					vs. pre-SplB w/o first methionine	vs. processed SplB
WT	24088	23939	22529	23957 > 24315 > 22687 > 24214	18	1428
E89G/S154R	24085	23936	22526	23956 > 24134 > 22687 > 24213	20	1430
N2K/S154R	24171	24022	22612	23956 > 24134 > 24217 > 22687 > 24297	66	1344
S154R	24157	24008	22598	24204 > 24026 > 23957 > 24284 > 23884 > 24303	196	1606

[a]*purified with N-terminal 6xHis-tag sequence: MGSSHHHHHHSQAS.* [b]*N-terminal "MGSSHHHHHHSQAS" sequence removed.* [c]*Sorted descending by signal intensity.* [d]*Calculated for highest intensity peak*

Supplementary Table 2: Crystallographic data summary for 6YV5 and 6YV6

Beamline	DIAMOND BEAMLINE I04	DIAMOND BEAMLINE I04
structure	SplB N2K/N3Q/S154R	SplB N2K/N3Q/S154R
Date of collection	13/09/17	13/09/17
pdb	6YV5	6YV6
**Data Collection**		
Wavelength	0.9795	0.9795
Resolution range	24.16 - 1.1 (1.139 - 1.1)	32.6 - 1.36 (1.40 - 1.36)
Space group	P 21 21 21	C 1 2 1
Unit cell	34.92 64.34 73.15 90 90 90	130.69 44.31 30.81 90 92.94 90
Total reflections	127080 (9031)	271636 (18425)
Unique reflections	66027 (5579)	37738 (2803)
Multiplicity	1.9 (1.6)	7.2 (6.6)
Completeness (%)	97.52 (83.54)	99.4 (99.4)
Mean I/sigma(I)	10.91 (1.29)	15.2 (1.1)
Wilson B-factor	12.48	18.65
R-merge	0.02728 (0.4075)	0.0052 (1.527)
R-meas	0.03858 (0.5763)	0.057(1.662)
R-pim	0.02728 (0.4075)	0.021 (0.641)
CC1/2	0.998 (0.665)	1.00 (0.56)
**Refinement**		
Resolution	24.16-1.10 (1.13-1.10)	19.36 - 1.36 (1.40-1.36)
Reflections used in refinement	66027 (5579)	37724 (2788)
Reflections used for R-free	3296 (299)	1912 (159)
R-work	0.186 (0.2303)	0.19 (0.2269)
R-free	0.20 (0.2369)	0.204 (0.2431)
Number of non-hydrogen atoms	1858	1743
macromolecules	1667	1594
Ligand		36
solvent	191	113
Protein residues	206	200
RMS(bonds)	0.01	0.013
RMS(angles)	1.48	1.7
Ramachandran favored (%)	96.57	97.45
Ramachandran allowed (%)	2.94	2.55
Ramachandran outliers (%)	0.49	0
Rotamer outliers (%)	0	0.58
Clashscore	3.33	1.22
Average B-factor	20.94	28.11
macromolecules	19.87	27
solvent	30.25	36.42
Ligands		51.04

Supplementary Table 3: DNA constructs used in this study

Name	Description
pCT-SplB-N3Q	Spl protease yeast display construct for non-native N-terminus (i.e. N-terminal Aga2 fusion)
pHAT5-SUMO-FDDAAD	Spl protease expression construct for E.coli for native N-terminus (pHAT5 is available from Addgene: https://www.addgene.org/112586/)
pHAT5-SUMO-FDDBAD	
pHAT5-SUMO-FDDBBD	
pHAT5-SUMO-FDDBCD	
pHAT5-SUMO-FDDBDD	
pHAT5-SUMO-FDDBED	
pHAT5-SUMO-FDDBFD	
pHAT5-SUMO-SplA	
pHAT5-SUMO-SplB	
pHAT5-SUMO-SplC	
pHAT5-SUMO-SplD	
pHAT5-SUMO-SplE	
pHAT5-SUMO-SplF	
pRSF-A2M-ENLYFQ-10xHis-Avi	A2M bait substrate expression construct for E.coli
pRSF-A2M-RWLLTbait-10xHis-Avi	
pRSF-A2M-VWLYSbait-10xHis-Avi	
pRSF-A2M-WELQbait-10xHis-Avi	
pRSFDuet-GST-Ulp1	SUMO protease co-expression plasmid (together with pHAT5 constructs)
pRSFNheI-SplB-N2K-N3Q-S154R	Spl protease expression construct for E.coli for N-terminal His-tag
pRSFNheI-SplB-N3Q-E89G-S154R	
pRSFNheI-SplB-N3Q-E89G	
pRSFNheI-SplB-N3Q-E89K	
pRSFNheI-SplB-N3Q-S23R-I66V	
pRSFNheI-SplB-N3Q-S154R	
pRSFNheI-SplB-N3Q	
pYD2-SplA-BsaI	Spl protease yeast display construct for native N-terminus, with BsaI stuffers pre-Golden Gate
pYD2-SplB-BsaI	
pYD2-SplC-BsaI	
pYD2-SplD-BsaI	
pYD2-SplE-BsaI	
pYD2-SplF-BsaI	
pYD2-SplB-N3Q-S157A	Spl protease yeast display construct for native N-terminus
pYD2-SplB-N3Q	
pYD2-SplFDDAAD	
pYD2-SplFDDBAD	
pYD2-SplFDDBBD	
pYD2-SplFDDBCD	
pYD2-SplFDDBDD	
pYD2-SplFDDBED	
pYD2-SplFDDBFD	

Supplementary Table 4: DNA oligonucleotides used in this study

Name	Description	Sequence
PK412	unbarcoded_fw_primer	GTCGATTTTGTTACATCTAC
PK421	unbarcoded_rev_primer	GCTTTGCCATTGGCCTTAGCTC
PK524	barcode_primer_rev_WELQS3h	AGCTCTGCAGTTGCGTCCTGTTACGAGAACTCATAAGGCTCTTTGGACAAGAG
PK496	barcode_primer_rev_RWLLT12h	AGCTCTGCAGAACTAGGCACAGCGAGTCTTGGTTAAGGCTCTTTGGACAAGAG
PK495	barcode_primer_rev_WELQS12h	AGCTCTGCAGTTCAGGGAACAAACCAAGTTACGTAAGGCTCTTTGGACAAGAG
PK494	barcode_primer_rev_VWLYS12h	AGCTCTGCAGGTGTTACCGTGGGAATGAATCCTTAAGGCTCTTTGGACAAGAG
PK493	barcode_primer_rev_RWLLT3h	AGCTCTGCAGTTCTCGCAAAGGCAGAAAGTAGTCAAGGCTCTTTGGACAAGAG
PK491	barcode_primer_rev_VWLYS3h	AGCTCTGCAGTTCGGATTCTATCGTGTTTCCCTAAAGGCTCTTTGGACAAGAG
PK490	barcode_primer_rev_RWLLT1h	AGCTCTGCAGGAGTCTTGTGTCCCAGTTACCAGGAAGGCTCTTTGGACAAGAG
PK489	barcode_primer_rev_WELQS1h	AGCTCTGCAGTCGATTCCGTTTGTAGTCGTCTGTAAGGCTCTTTGGACAAGAG
PK488	barcode_primer_rev_VWLYS1h	AGCTCTGCAGAAGAAAGTTGTCGGTGTCTTTGTGAAGGCTCTTTGGACAAGAG
PK464	PstI-Barcode-ins-fw	GCGGTTCTCACCCCTCAACAACTAGCAAAGGCAGCCCCATAAACACACAGTATGTT TTTTAACTCGAGNNNNNNNNNNCTGCAGATCTGATAACAACAGTGTAG
PK463	HR-PstI-ins-rev	ACGACGGCCAGTGAATTGTAATACGACTCACTATAGGGCGAATTGGAGCTCAATTC TCTTAGGATTCGATTC
PK422	ep1st-pYD2-rev	CTAAAGTTGGTGAGGGGATTTGCTCG
PK418	ep1st-pCT-rev	GTTGTTATCAGATCTCGAGCTATTACAAGTCC
PK417	ep1st-pCT-fw	CGACGATTGAAGGTAGATACCCATACG

Uncropped scans of all and gels

For details, please refer to the caption of the corresponding Supplementary Figure.

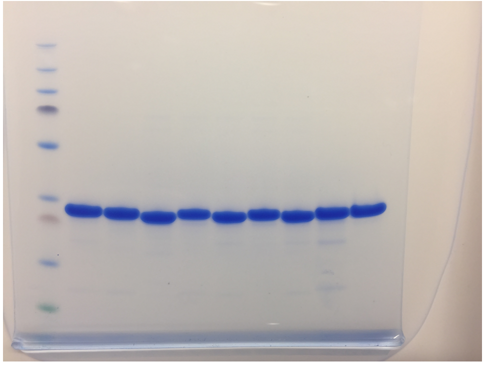

SDS PAGE gel shown in Supplementary Figure 2b.

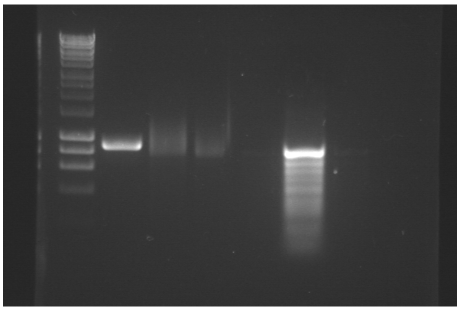

Agarose gel shown in Supplementary Figure 4d.
