# Supplementary material for: Evolution of protease activation and specificity via alpha-2-macroglobulin-mediated covalent capture
Source: Nat Commun. 2023 Feb 11;14:768. doi: 10.1038/s41467-023-36099-7 (PMC9918453; doi:10.1038/s41467-023-36099-7)
Supplement: Supplementary file 2 — Reporting Summary [file 41467_2023_36099_MOESM2_ESM.pdf]

nature research

Corresponding author(s): Florian Hollfelder

Last updated by author(s): 18/12/22

Reporting Summary

Nature Research wishes to improve the reproducibility of the work that we publish. This form provides structure for consistency and transparency in reporting. For further information on Nature Research policies, see our Editorial Policies and the Editorial Policy Checklist.

Statistics

For all statistical analyses, confirm that the following items are present in the figure legend, table legend, main text, or Methods section.

n/a	Confirmed	
☐	☒	The exact sample size (*n*) for each experimental group/condition, given as a discrete number and unit of measurement
☐	☒	A statement on whether measurements were taken from distinct samples or whether the same sample was measured repeatedly
☒	☐	The statistical test(s) used AND whether they are one- or two-sided<br>*Only common tests should be described solely by name; describe more complex techniques in the Methods section.*
☒	☐	A description of all covariates tested
☒	☐	A description of any assumptions or corrections, such as tests of normality and adjustment for multiple comparisons
☐	☒	A full description of the statistical parameters including central tendency (e.g. means) or other basic estimates (e.g. regression coefficient) AND variation (e.g. standard deviation) or associated estimates of uncertainty (e.g. confidence intervals)
☒	☐	For null hypothesis testing, the test statistic (e.g. *F*, *t*, *r*) with confidence intervals, effect sizes, degrees of freedom and *P* value noted<br>*Give P values as exact values whenever suitable.*
☒	☐	For Bayesian analysis, information on the choice of priors and Markov chain Monte Carlo settings
☒	☐	For hierarchical and complex designs, identification of the appropriate level for tests and full reporting of outcomes
☒	☐	Estimates of effect sizes (e.g. Cohen's *d*, Pearson's *r*), indicating how they were calculated

Our web collection on statistics for biologists contains articles on many of the points above.

Software and code

Policy information about availability of computer code

Data collection

- Absorbance and Fluorescence Data with Tecan Infinite 200 PRO plate reader: Tecan i-control 1.10;
- FACS data with a Becton Dickinson FACScan Cytek DxP
- X-ray diffraction data were collected at the Diamond Light Source beamlines I04;
- Fastq-files were generated using a MinION using high accuracy (HAC) base calling setting in MinKNOW. Input library sequencing data obtained from three different runs were combined.

Data analysis

The following software was used in this study:
- FlowJo v10.1
- GraphPad Prism 5.0c
- XDS and XSCALE, autoproc ; (in diamond autoprocessing)
- Coot 0.9.5
- Aimless 0.7.0 (in diamond autoprocessing)
- Buccaneer 1.16.9
- Phaser 2.1.2 and Refmac5 5 (CCP4 suite 7.1.015)
- Dials 2.0 (diamond autoprocessing)
- Grade 1.2.20 (Global Phasing)
- PyMOL Version 1.7.6.0
- NanoFilt (github.com/wdecoster/nanofilt)
- Nucleotide-Nucleotide BLAST 2.7.1+
- INC-seq (https://github.com/CSB5/INC-Seq)
- seqkit 0.12.0 (https://github.com/shenwei356/seqkit)
- SCHEMA-RASPP (https://github.com/mattasmith/SCHEMA-RASPP)
- Pandas 0.24.2 for Python 2.7
- Python 2.7.16

- Seaborn 0.9.0 for Python 2.7

For manuscripts utilizing custom algorithms or software that are central to the research but not yet described in published literature, software must be made available to editors and reviewers. We strongly encourage code deposition in a community repository (e.g. GitHub). See the Nature Research guidelines for submitting code & software for further information.

Data

Policy information about availability of data

All manuscripts must include a data availability statement. This statement should provide the following information, where applicable:

- Accession codes, unique identifiers, or web links for publicly available datasets
- A list of figures that have associated raw data
- A description of any restrictions on data availability

Data availability statement

The crystallographic data is deposited at RCSB PDB under accession numbers:
- 6YV5 [https://www.rcsb.org/structure/6YV5] (SplB N3Q/S154R)
- 6YV6 [https://www.rcsb.org/structure/6YV6] (SplB N2K/N3Q/S154R)

Public datasets used are available under:
- 2VID [https://www.rcsb.org/structure/2VID] (SplB)
- 4K1S [https://www.rcsb.org/structure/4K1S] (GS-SplB)

Sequence files of DNA constructs used in this study can be found online at Benchling [https://benchling.com/philipp_knyphausen/f_/M2tyfz1K-a2m_deposit/].

The following previously published structures are available

The following figures have associated source data:
Figs 1c, 2b, 2c, 2e, 3b, 4e, 5b, 5c, 5e, 6a
Suppl. Fig. 1b, Suppl. Fig.4, Suppl. Fig. 6

The sequence run data is deposited at the European Nucleotide Archive under :
ERX9375390 (input library)
ERX9375391 (output library)

The processed sequencing data are available in the Source Data file accompanying this paper.

Field-specific reporting

Please select the one below that is the best fit for your research. If you are not sure, read the appropriate sections before making your selection.

☒ Life sciences ☐ Behavioural & social sciences ☐ Ecological, evolutionary & environmental sciences

For a reference copy of the document with all sections, see nature.com/documents/nr-reporting-summary-flat.pdf

Life sciences study design

All studies must disclose on these points even when the disclosure is negative.

Sample size	- Culture volumes and sample sizes of yeast cells were adjusted to ensure an at least ten-fold excess of cells relative to the library diversity (OD600 1 ~ 3 × 10^7 cells/ml).<br>- For MACS of the epPCR library, ~1 × 10^9 cells were used and ~1x10^7 magnetic beads were used.<br>- The second round of the epPCR library and the shuffling library selections were carried out with ~1 × 10^7 yeast cells and ~1 × 10^6.<br>- Sample sizes for flow cytometry and in vitro kinetic analysis were chosen based the magnitude of measurable differences and the consistency of the measurements.
Data exclusions	See methods section and the NGS analysis scripts in the Source Data for the full filtering steps applied in the processing of the NGS dataset. In the nanopore sequencing results, reads of Phred score <9 were discarded. INC-seq reads with less than 3 repeats excluded from consensus sequence generation.
Replication	Flow cytometry of yeast populations including labeling showed high degrees of reproducibility across independent inductions. Sample sizes for each experiment are indicated in the figure legends.<br><br>Michaelis-Menten parameters were derived from multiple datapoints that collectively also suggest acceptable reproducibility. For the determination of Michaelis-Menten-Kinetics, the requirement for a calibration curve and background substraction is pointed out in the manuscript. Sample sizes for each experiment are indicated in the figure legends.
Randomization	Randomization was not relevant to this study as samples were not grouped for experiments or analyses.

Blinding	Blinding was not relevant to this study as samples were not grouped for experiments or analyses.

Reporting for specific materials, systems and methods

We require information from authors about some types of materials, experimental systems and methods used in many studies. Here, indicate whether each material, system or method listed is relevant to your study. If you are not sure if a list item applies to your research, read the appropriate section before selecting a response.

Materials & experimental systems

n/a	Involved in the study
☐	☒ Antibodies
☒	☐ Eukaryotic cell lines
☒	☐ Palaeontology and archaeology
☒	☐ Animals and other organisms
☒	☐ Human research participants
☒	☐ Clinical data
☒	☐ Dual use research of concern

Methods

n/a	Involved in the study
☒	☐ ChIP-seq
☐	☒ Flow cytometry
☒	☐ MRI-based neuroimaging

Antibodies

Antibodies used	- Streptavidin-PE (1:200, Biolegend, cat. no. 405204)<br>- PE anti-streptavidin (1:200, Biolegend, cat. no. 410503, clone 3A20.2<br>- anti-c-myc-FITC (1:50, Miltenyi Biotec, cat. no. 130-116-485, clone SH1-26E7.1.3)
Validation	Antibodies were used without validation.<br><br>Supplier notes on quality control:<br>- Streptavidin-PE:<br>This streptavidin product is quality control tested by immunofluorescent staining with flow cytometric analysis.<br><br>- PE anti-streptavidin:<br>Isotype Control: PE Mouse IgG2b, κ Isotype Ctrl<br>Antibody Type: Monoclonal, Host Species: Mouse, Immunogen: Streptavidin<br>Each lot of this antibody is quality control tested by immunofluorescent staining with flow cytometric analysis.<br><br>- c-myc Antibody:<br>anti-human/mouse/rat<br>Isotype:mouse IgG1<br>Applications:FC, IF, WB<br>Tested: QC tested

Flow Cytometry

Plots

Confirm that:

☒ The axis labels state the marker and fluorochrome used (e.g. CD4-FITC).

☒ The axis scales are clearly visible. Include numbers along axes only for bottom left plot of group (a 'group' is an analysis of identical markers).

☐ All plots are contour plots with outliers or pseudocolor plots.

☐ A numerical value for number of cells or percentage (with statistics) is provided.

Methodology

Sample preparation	Cells were washed with 200 µl PBS/0.5% BSA
Instrument	Becton Dickinson FACScan Cytek DxP machine with a 488 nm laser / 530/30 nm filter for FITC and a 561 nm laser / 590/20 nm filter for PE.
Software	FlowJo version 10.6.1
Cell population abundance	The number of the original cell population and the final percentages of cells that were analyzed are summarized in the

Cell population abundance	Source Data for each flow cytometry plot (data is provided for Fig. 1c, Fig. 2b, c, e, Fig. 4e, Fig. 5c, Fig. 6a, Suppl. Fig. 1b, Suppl. Fig. 4, Suppl. Fig. 6).
Gating strategy	Yeast cells were gated based on FSC/SSC. An examplary gating plot is shown in Suppl. Fig. 1b.

☒ Tick this box to confirm that a figure exemplifying the gating strategy is provided in the Supplementary Information.
